# Gamifying Breastfeeding for Fathers: Process Evaluation of the Milk Man Mobile App

**DOI:** 10.2196/12157

**Published:** 2019-06-20

**Authors:** Becky White, Roslyn C Giglia, James A White, Satvinder Dhaliwal, Sharyn K Burns, Jane A Scott

**Affiliations:** 1 School of Public Health Curtin University Perth Australia; 2 Telethon Kids Institute University of Western Australia Perth Australia; 3 Reach Health Promotion Innovations Perth Australia; 4 Collaboration for Evidence, Research and Impact in Public Health Curtin University Perth Australia

**Keywords:** mHealth, app, breastfeeding, fathers, gamification, socially connected, push notifications

## Abstract

**Background:**

Mobile technology offers unique opportunities to reach people with health promotion interventions. Breastfeeding is an important public health issue, and fathers are a key support. Milk Man is a father-focused breastfeeding app that sought to engage fathers with information and conversation about breastfeeding, with the goal to impact positively on breastfeeding duration.

**Objective:**

The study aimed to describe the process evaluation of the Milk Man app that was trialed in the Parent Infant Feeding Initiative randomized controlled trial.

**Methods:**

The app used an information library, gamification, push notifications, and social connectivity via a Web-based conversation forum, which included polls and conversation starters, to engage fathers with breastfeeding information. Fathers had access to the app from approximately 32 weeks of gestation to 6 months postpartum. Process evaluation data were collected from a self-completed questionnaire administered via a Web-based link sent to participants at 6 weeks postpartum, and app analytics data were collected directly from the app. Quantitative data from both sources and qualitative responses to open-ended questions were used to triangulate findings to investigate patterns of usage and the effectiveness of each app engagement strategy to motivate and engage users.

**Results:**

A total of 80.3% (586/730) of participants, who were randomized to receive the app, downloaded Milk Man. Push notifications and interest in what other fathers had posted in the forum were the 2 main motivators to app use. Fathers used the app most while their partners were still pregnant and in the weeks immediately after the birth of their baby. Perspectives on the gamification strategy were varied. However, at 6 weeks postpartum, approximately one-third of fathers still using the app said that the gamification elements were encouraging the app use. The ease of use of the app and the design were important elements that were rated positively. The conversation forum emerged as the hub of app activity; all but 1 of the most accessed library articles and external organization links had been prompted as part of a conversation starter. Fathers posted comments in the conversation forum 1126 times (average of 2.21 per user) and voted in polls 3096 times (average of 6 per user).

**Conclusions:**

These results demonstrate that the Milk Man app was an acceptable source of breastfeeding information and support that fathers and fathers-to-be are prepared to use throughout the perinatal period. The app showed encouraging results with facilitating conversation between partners. The conversation forum was clearly central to the success of the app, and fathers provided suggestions for improvement. Gamification results were varied, yet it was a key motivator for some users. These results provide valuable insight into the acceptability of the engagement strategies, including motivations for use and user perspectives on the app.

**Trial Registration:**

Australian New Zealand Clinical Trials Registry ACTRN12614000605695; https://www.anzctr.org.au /Trial/Registration/TrialReview.aspx?ACTRN=12614000605695

## Introduction

### Breastfeeding

Breastfeeding is a key public health issue with well-evidenced health benefits for both infants and mothers [1,2]. For infants, short-term benefits include protection from gastrointestinal and respiratory tract infections, otitis media, and sudden infant death syndrome [3-5], and long-term benefits include a lower risk of obesity and type 2 diabetes [6]. For mothers, benefits include a reduction in the risk of ovarian and breast cancer, diabetes, and hypertension [2,7]. The World Health Organization recommends exclusive breastfeeding for the first 6 months of life, with breastfeeding to continue thereafter with the introduction of complementary foods [8]. Despite near-universal initiation of breastfeeding among Australian women [9], the latest available data (2014-2015) from the National Health Survey indicate that only one-quarter (24.7%) of infants are exclusively breastfed to at least 6 months [10], and less than 6 out of every 10 infants receive any breast milk at the age of 6 months [9]. These statistics have remained relatively stagnant for the last two decades or so [11], and new and innovative ways of increasing the duration and exclusivity of breastfeeding are urgently needed to ensure that most Australian children (and their mothers) receive maximum and continued benefits of breastfeeding.

### Social Support and Breastfeeding

Although there are many complex factors that can impact on breastfeeding [12,13], social support is one of the most crucial [14]. In Western societies in particular, there is convincing evidence that fathers are a key source of breastfeeding support and influence decisions regarding both the initiation [15,16] and duration [17,18] of breastfeeding and contribute to maternal breastfeeding confidence [19,20]. Due to the value of this support, much research has been conducted to define what contributes to positive paternal support, and how health professionals can better support fathers [21-23]. Previous empirical and qualitative studies tells us that fathers want the following: more education to feel more empowered about their role in breastfeeding, increased social support, and help to overcome specific barriers (including public breastfeeding and perceived bonding postponement) [22-25].

However, while they are encouraged to, and often do, attend antenatal classes with their partners, these classes are generally directed at the mother, and men feel left out or feel that their role and their information and support needs are not a priority [26]. Furthermore, work commitments may limit a man’s involvement in their partner's pregnancy care and the number of antenatal classes and appointments that they can attend [27]. Information and support, therefore, needs to be targeted toward men in a way that is appropriate and readily accessible [27].

### Mobile Technology and Health Promotion

Electronic and mobile technology offers public health researchers unique opportunities to reach people with health information and tailored interventions with a wide reach and at a low cost. Parents have traditionally accessed the internet for information on pregnancy and early parenting [28,29], but newer digital media information sources, such as apps and social media platforms, are increasingly being used [28,30]. Men are seeking information about parenting and infant care (including breastfeeding), supporting and improving their relationship with their partner, and managing stress [29]. They are accustomed to ready and immediate access to information using digital technologies and want better access to information than that offered by health professionals [28]. Mobile technology can provide the user with accessible information despite geographical distance or time constraints, and the immediacy of this technology provides users with information when it is most needed [28]. Peer support can be provided through app-based and Web-based forums and can assist the transition to fatherhood by providing fathers with the opportunity to share information and experiences, mutual support, and the recognition that they are not alone with their problems [31,32].

Although digital technologies, including mobile apps, targeted at mothers have been used successfully to improve breastfeeding outcomes [33], there were no digital technology–based breastfeeding interventions specifically targeting fathers at the time this research was conceived. Smartphone ownership is almost universal (89%) among Australian adults across men and women [34]. The Milk Man app was conceived as a novel way of delivering targeted breastfeeding information to fathers in a readily accessible format. Little was known about how fathers would receive and use such an app. There is a growing consensus that the concept of engagement in digital health interventions encompasses a range of metrics including both usage and reported experience [35]. This paper adds to the evidence by describing the process evaluation of the Milk Man app, investigating which of the app engagement strategies were effective in motivating and engaging users in app use by using a combination of data from the app analytics framework as well as self-report data from a questionnaire.

## Methods

### The Milk Man App

Milk Man was a mobile app designed to provide fathers with information and support about breastfeeding and was developed to be trialed in the Parent Infant Feeding Initiative (PIFI), a 4-armed factorial randomized controlled trial (RCT; ACTRN12614000605695) [36]. The PIFI study aimed to examine the impact on breastfeeding duration of 2 separate father-focused breastfeeding interventions (a male-facilitated, father-focused, antenatal breastfeeding class and the Milk Man app) both in isolation and in combination, compared with a control group that received usual care.

The app’s design and development have been described previously [37]; in brief, Milk Man was developed based on a best practice approach [38] that involved development by a multidisciplinary team, in consultation with new and expecting fathers and was based on the social cognitive theory [39]. A range of sophisticated engagement strategies, designed to encourage fathers to start and continue using the app, were employed. The app contained a comprehensive evidence-based information library presented in a colloquial and light-hearted manner. The library included information about a wide variety of breastfeeding-related topics and broader parenting topics, posted by the research team, that were designed to encourage fathers to read the information in the library. A list of the library headers has been included in Multimedia Appendix 1.

**Figure 1:**
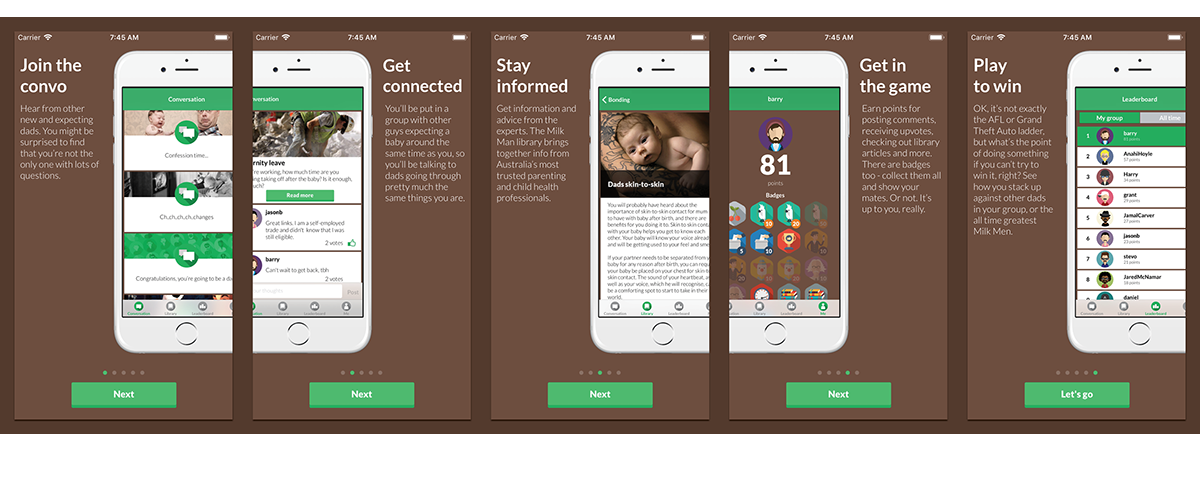
Milk Man onboarding screenshots.

Fathers were placed into a group with others at a similar perinatal stage to facilitate relevant conversations. The conversation also used polls comprising multiple-choice questions, where users could choose an answer and view the aggregated responses of other users. Biweekly push notifications were used to alert the fathers to new content being added to this conversation and to remind them to check-in. The notifications were sent around lunchtime, and it read as follows: *there’s a new conversation starting*. Users could swipe the notification to be taken directly to the new content in the conversation. The conversation forum was monitored by researchers throughout the study [31]. The app’s engagement strategy was underpinned by gamification. Gamification elements, such as points, badges, and leaderboards, were integrated into the app, and fathers received virtual rewards in the form of points for completing the actions the researchers wanted to encourage, such as reading articles and commenting on forum posts. A 2-tiered leaderboard system was introduced whereby participants could see their position on the leaderboard, both within their own group and within the whole cohort. Figure 1 shows the introductory onboarding screens shown to users when they first opened the Milk Man app, explaining the various components of the app, including the library, conversation, points, badges, and leaderboards.

### Participants

Participants were recruited directly by members of the research team from hospital-based antenatal classes in metropolitan Perth, Western Australia, between August 2015 and December 2016. Couples were eligible to participate if they owned a compatible smartphone (iOS or Android), lived in Western Australia, had internet access, spoke English, and if both parents intended to coparent their child. Signed informed consent was obtained face to face from both fathers and mothers at the time of recruitment. The study was approved by the Curtin University Human Research Ethics Committee (HR 82/2014; May 14, 2014).

### Study Design

Fathers randomized into either of the 2 intervention groups that had access to the Milk Man app were provided with instructions on how to access the app. There was no prescribed usage; participants were asked to use the app as they would use any other app. Fathers had access to Milk Man from when they signed up for the study (at an average of 32.5 gestational weeks) to 26 weeks postpartum. Questionnaire data were collected at recruitment and at 6 and 26 weeks postpartum. Preliminary analysis of the data revealed that app usage was the highest in the first 6 weeks and declined thereafter and that little new information was obtained from the 26 weeks questionnaire. Hence, this study reports results to 6 weeks postpartum.

Process evaluation describes the implementation of an intervention and seeks to understand how the study functioned and how participants reacted [40]. A comprehensive evaluation plan was developed for the study that has been described previously [41]. On the basis of a framework developed by O’Grady et al [42], the evaluation plan outlined evaluation indicators over 5 distinct focus areas: *people, content, technology, computer-mediated technology,* and *health system integration*. The results of the process evaluation are presented according to these 5 areas of focus that are briefly defined below:

- People: Fathers’ perspectives on the app including intentions and motivators for use and satisfaction with the app.
- Content: The library content built into the app and the dynamic user-generated content in the conversation forum. User’ perspectives on the key engagement strategies are also included in this section including gamification and push notifications.
- Technology: This refers to describing and monitoring the software that was created to run the app, including tracking how participants used the app and the response of the software to operating system updates for the duration of the trial.
- Computer-mediated technology: This refers to the interaction of the users and the app interface and whether this supported community interaction. It includes examining how easy it was for participants to locate information and the usability of the app and the user perspectives on the app in general.
- Health system integration: The impact the app had on the participants’ use of other services. These were measured through app-originated visits to external service provider’s websites.

Quantitative evaluation data were collected from 2 different sources, a custom-built app analytics framework and a Web-based questionnaire, the link to which was sent to fathers via an email at 6 weeks postpartum. The analytic framework was embedded in the Milk Man app, and it recorded user actions performed in the app over time. These actions included app opens, the article reads, and engagement in the gamification and conversation forum. The framework allowed for more fine-grained analysis and data matching as compared with other commercially available frameworks and was integral to the ongoing monitoring of the robust process evaluation indicators.

The questionnaire sought the fathers’ perspectives of the Milk Man app. The questions were specific for this study and were guided by the key items that have been identified as important in app quality—engagement, functionality, aesthetics, information, and personal opinion [43]. Most questions required respondents to indicate on a 5-point Likert scale the degree to which they agreed or disagreed with a statement related to the usability and acceptability of the app, with some additional questions presented as an open text response or a multiple-choice question. To reduce participant burden, none of the questions in the questionnaire were compulsory to answer, and the denominators presented in this paper show the actual number of respondents to each individual question. Qualitative responses to open-ended questions are used as quotes to illustrate the sentiments of app users. The questions asked about the Milk Man app are included as Multimedia Appendix 2.

## Results

### Participant Characteristics

**Figure 2:**
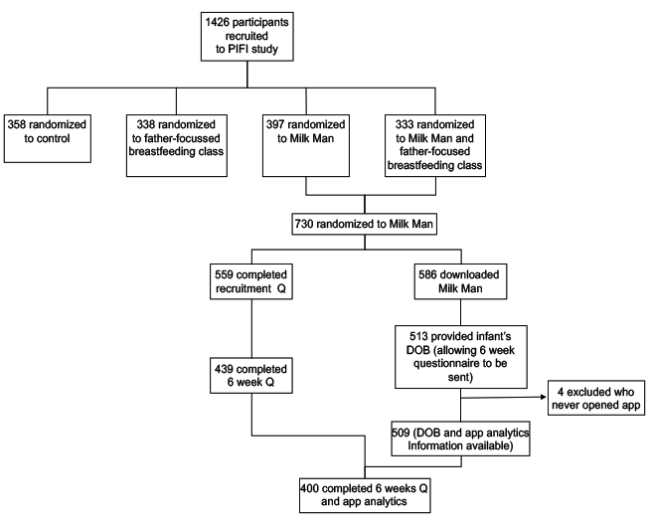
Participant flowchart. PIFI: Parent Infant Feeding Initiative; DOB: date of birth; Q: Questionnaire.

A total of 1426 couples were recruited to the RCT, with 51.19% (730/1426) of couples being assigned to an intervention group with access to Milk Man. Of these, 80.3% (586/730) of the fathers downloaded the Milk Man app, providing app analytics data. The fathers were asked to provide the date of birth of their baby, and this was needed to enable the 6-week questionnaire to be sent out and for mapping of the analytics over time. A total of 76.6% (559/730) and 60.1% (439/730) of the fathers completed the baseline and the 6-week questionnaires, respectively. Figure 2 shows the participant flow in the study.

A summary of baseline demographics for those with access to Milk Man is presented in Table 1. The median age of fathers (33.0 years) was similar to that of Australian fathers of newborn children (33.3 years) [44]. Most of the PIFI cohort (66.2%) were born in Australia, which also mirrors the general population (67%) [45]. However, they were more highly educated than the general population as only 25.4% of Australian men aged between 20 and 39 years have completed a Bachelor’s degree or higher [46].

### Process Evaluation Indicators

For clarity of reporting, throughout the Results section, participants are referred to as *respondents* when data derived from the questionnaires completed at 6 weeks are reported, and as *users* when data collected from the app analytics framework are reported.

**Table 1:** App group participant demographic characteristics.

Characteristic		Parent Infant Feeding Initiative, n (%)
**Age (years; n=559)**		
	<30	97 (17.3)
	30-34	249 (44.5)
	35+	213 (38.1)
**Education (n=550)**		
	High school or trade certificate	201 (36.5)
	Undergraduate university education or higher	349 (63.5)
**Country of birth (n=551)**		
	Australia/New Zealand	365 (66.2)
	United Kingdom/Eire	69 (12.5)
	Africa/Middle East	39 (7.1)
	Asia	39 (7.1)
	Other	39 (7.1)

#### People

**Figure 3:**
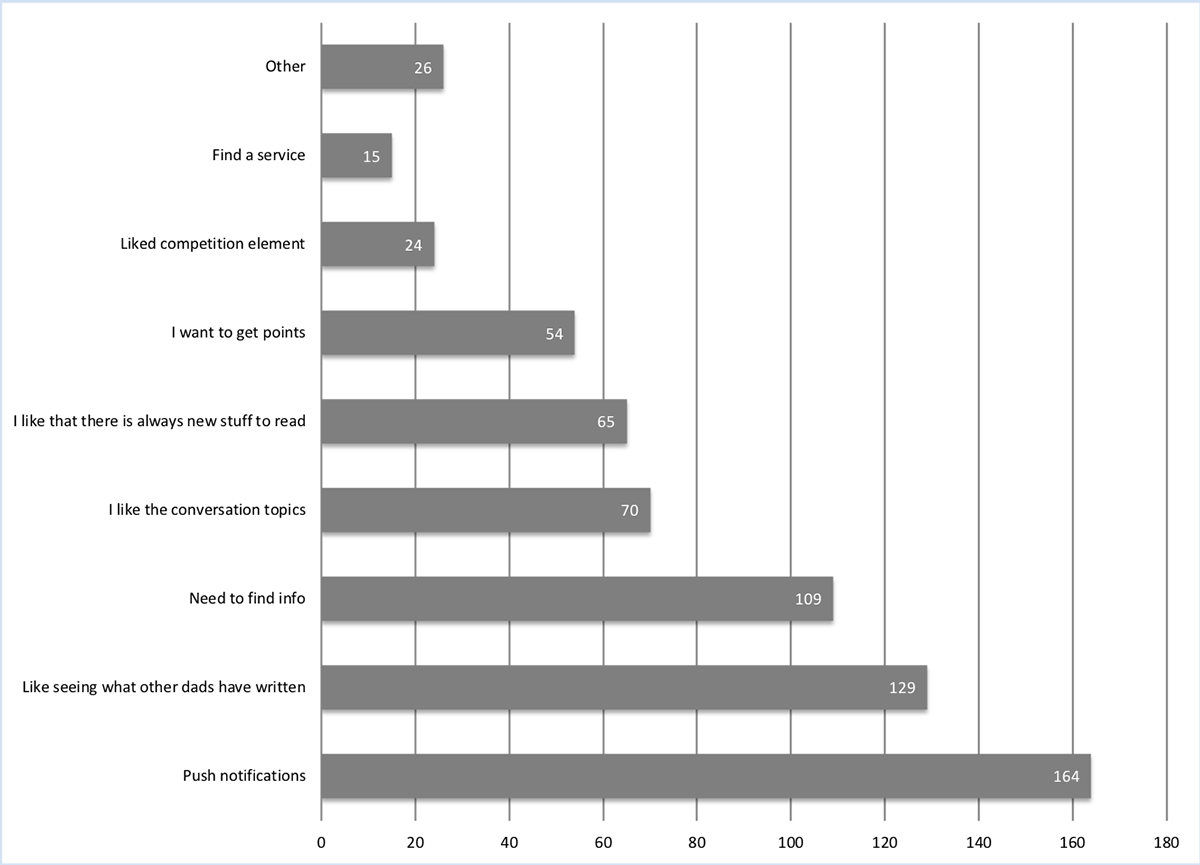
Motivators to use app (respondents could choose more than one response).

The majority of fathers responding to the 6-week questionnaire (367/390, 94.1%) indicated that they had downloaded the Milk Man app. The most common reasons given by respondents for not downloading the app were either *too busy* or *just not gotten around to it*. Motivators for using the Milk Man app are described in Figure 3. Push notifications were the highest reported motivating factor (n=164). This was followed by liking seeing what other dads had written (n=129) and the need to find information (n=109).

Overall, respondents’ perspectives on the app were positive. In total, 247 of 296 responders (83.4%) agreed that the app was easy to use, and 231 of 296 responders (78.0%) said the visual design was appealing. Two-thirds of respondents would recommend the app to other fathers (199/296), and 59.0% of respondents agreed that it was interesting or fun to use (170/288). In terms of actual breastfeeding support, 54.6% of respondents (161/295) agreed that the app had made them more aware of how they could help with breastfeeding, and a similar proportion of respondents (160/296, 54.0%) indicated that the app had led to discussions with their partner.

#### Content

##### Information Library

App analytics data showed the range of library articles viewed by users was 0 to 79 (mean 11.46 per participant, SD 13.7). All except 1 of the most frequently accessed articles were linked to via a conversation topic. Many of the library articles contained links to external sources including websites and YouTube videos. Users followed unique links to external sites (not including multiple visits to the same link over time) between 0 and 43 times. The average number of unique links followed per person was 3 (SD 5.3). The top 10 most followed links were all associated with topics in the conversation forum, either by a direct link from the topic or from a library article the topic linked to.

Overall, responses to the 6-week questionnaire, regarding perspectives on the library, were positive and demonstrated value to respondents. Two-thirds of respondents reported that they found the information easy to find (201/297, 67.7%) and that the links were appropriate and useful (194/296, 65.5%). The following comments from the 6-week questionnaire reinforce these results:

The information was useful and especially links to other websites and organisation[s].

It’s helpful to have information at your fingertips.

Informative, fun and covers different areas of breastfeeding.

A total of 72.3% of respondents (214/296) said that they learned new information from the library, whereas 78.4% of respondents (233/297) trusted the information. However, only 23.6% of respondents (70/296) reported using the app when they needed to find information, and 57.6% of respondents (171/297) agreed that the library contained enough information. The following comments were received from the fathers in the 6-week questionnaire:

Maybe more content in the LIBRARY that doesn't necessarily focus as much on breastfeeding but on other newborn baby facts/issues/problems/events.

It’s actually very good like it is. More contents would be good though.

##### Conversation

The total number of comments posted in the conversation forum by users was 1126. The number of comments made by each participant ranged from 0 to 57 with an average of 2.21 (SD 5.246). The fathers used the conversation to offer and seek social support, to source connection and information, and to share experiences. Users voted on polls 3096 times.

The fathers were asked their perspectives on the conversation section of the app. The responses to each of the questions in the 6-week questionnaire are displayed in Table 2. Overall, 63.0% of respondents (186/295) agreed (choosing to agree or strongly agree) that it was good to hear from other dads; however, only 29.9% (89/297) agreed that they found the conversation engaging. In addition, 37.1% of respondents (110/296) reportedly returned to the conversation after first viewing the topic to see if there were any new comments in the thread.

Respondents to the 6-week questionnaire were also asked about the impact the app had on generating conversations. A total of 54.1% (160/296) said that the information in the app had led to conversations with their partner, and 52.5% (156/297) said that the conversation forum itself had prompted discussions. These results were more pronounced for those who used the app for a longer period. Of those respondents who had stopped using Milk Man before 6 weeks postpartum, 38.3% (54/141) said that the conversation forum had prompted a discussion with their partner, compared with 65.4% (102/156) who were still using it at 6-weeks postpartum. Similarly, only 34.3% (48/140) of respondents not using the app at 6 weeks postpartum said that the information in the app had prompted a discussion, compared with 71.8% (112/156) who were still using the app. Respondents also provided comments indicating that the app was raising new information:

[Good for] generating discussion for something not normally considered.

[I liked] Different topics provided that fathers may not have thought to discuss or read up on.

Due to the way fathers were grouped depending on when their baby was due, the sizes of some of the conversation groups were quite small (group numbers ranged from 16 to 47), and the small numbers in some groups impacted the level of conversation. Several of the fathers noted that having an active researcher participating in the conversation could be of benefit:

It’s pretty quiet in there, hardly any interaction to comments. Need to get someone in there to reply to comments, get things going a bit in there.

The community is either not big enough or I am limited to only being exposed to what my own group posts. I find most of the time the conversation sections are empty. I post something and rarely does anyone else respond. I am 11th in the leaderboard and feel I have barely contributed. The people above me I have basically never seen post so maybe they used it a while ago and have since stopped?

Other suggestions for increasing collaboration included incorporating threaded replies and increasing the number of polls. A total of 4 fathers suggested incorporating a face-to-face aspect would be beneficial as well:

I think the app would work better if you had met the other dads a few times.

A real-world meetup would be nice as well - over a couple of beers.

The conversation was also one of the most commonly cited aspects that the fathers liked about the app. Some fathers reported that the conversation had helped them feel less alone and had created a sense of community. Others reported enjoying the polls, talking to others, and the humor:

Hearing from other dads; the community feel.

Helpful tips from other blokes who are in the same position.

It’s a reminder that I'm not alone!

**Table 2:** User perspectives on the conversation.

User perspectives on Milk Man Conversation Forum	Agree or strongly agree, n (%)	Neither agree nor disagree, n (%)	Disagree or strongly disagree, n (%)
I find the conversation engaging (n=297)	89 (29.9)	125 (42.1)	83 (27.9)
It was good hearing from other dads (n=295)	186 (63.0)	84 (28.5)	25 (8.5)
I sometimes returned to the conversation to see if there were any new comments (n=296)	110 (37.2)	88 (29.7)	98 (33.1)
I trusted the information in the conversation (n=297)	89 (29.9)	170 (57.2)	38 (12.8)
I have acted on advice that I have read in the conversation (n=297)	57 (19.2)	149 (50.1)	91 (30.6)

##### Push Notifications

**Figure 4:**
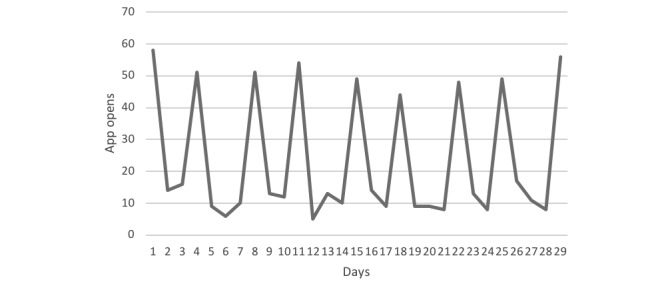
App usage over a 1-month period.

The most common thing that motivated the responders to the 6-week questionnaire to use the app was receiving the biweekly push notifications. This is reinforced by app analytics data on the usage of the app showing consistent spikes in activity on the days new content was added to the conversation and the push notifications were sent out. The usage over a 1-month period is displayed in Figure 4, demonstrating consistent spikes in activity on the days push notifications were sent out. This usage was typical of what was observed throughout the study. The average number of times that users swiped into the app from the push notifications was 2.5, and this indicated that although the swipe function was not highly used, the push notifications were a trigger for app use.

##### Gamification

Users earned points for their level of participation with the different components of the app. The number of points achieved by users ranged from 0 to 153 with an average of 22.24 per user (SD 25.6). Badges were another feature of the gamification strategy and were earned for completing certain actions. The most commonly achieved badges were as follows: voting on 5 polls (n=231), reading 10 articles (n=195), posting their first comment (n=187), opening the app 5 weeks in a row (n=184), and voting on 10 polls (n=155).

For those who were still using the app at 6-weeks postpartum, approximately one-third of respondents said that the gamification elements were encouraging that use. This included earning points (64/156, 41.0%), earning badges (54/156, 34.6%), and their position on the leaderboard (43/154, 27.9%). Those who had stopped using the app before completing the 6-week questionnaire were significantly less likely to agree that any of the gamification functions encouraged their use (*P*<.001).

There was a diverse range in respondents’ opinions on the gamification. Some fathers reported enjoying the gamification elements and said that aspects of it actively encouraged their continued use of the app, with some reporting that it was their main motivator:

Have you seen my points? I'm totally kicking ass.

[I liked] the competition aspect.

Others, however, did not like it and some respondents reported that it discouraged their use of the app. The following comments were posted in response to the open-ended questions asking what respondents liked about the app, and what could improve it:

Make it a little easier to earn points and badges, at least initially, to motivate use.

Review the points system as having points for people liking your comments etc creates scenarios of people making comments for the sake of it to get points.

Change out the leaderboard style for one where people earn status credentials, where people's credentials are listed next to their name on posts. E.g. such as how's it is done with reviewers in Amazon. Personally I do not want to be listed on a leaderboard on this kind of app; it didn't encourage me to use the app.

#### Technology

**Figure 5:**
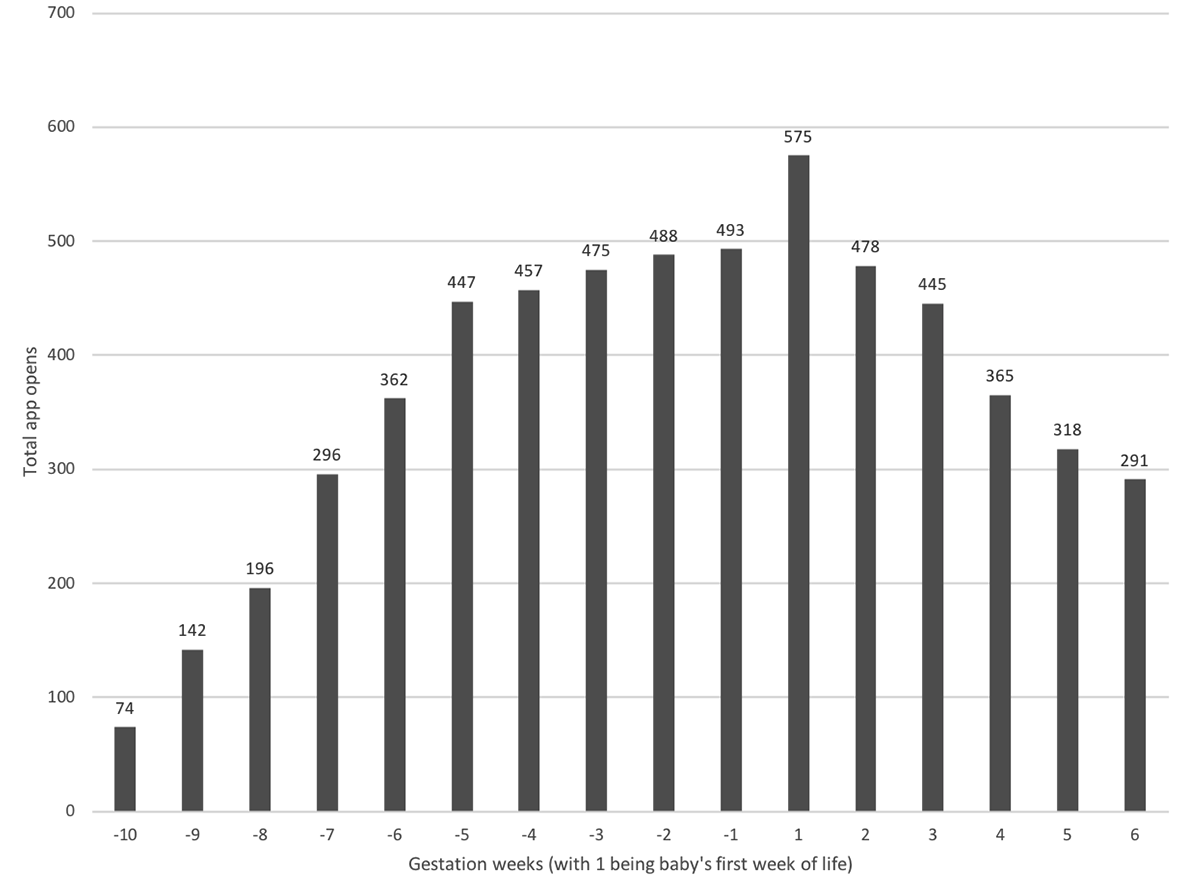
Unique days app was opened over time.

The app was built for the iOS and Android platforms and included a customized app analytics framework that tracked how and when individual fathers were using the app over time. Figure 5 shows the aggregated total number of unique days the app was opened each week, ranging from 10 weeks before birth, to 6 weeks after the birth of their baby. The graph shows that the highest usage of the app by fathers was in the week their baby was born.

During the implementation of the study (24 months), there were 4 operating system updates (2 iOS and 2 Android), and the app required updating a total of 4 times. A detailed log was kept of each technological event that happened over the trial period and the impact it had on the app intervention and app users. There were 2 major technological events that had an impact on the app during the intervention. The first was the retiring of the Parse service that was hosting the backend of the app, resulting in the need to migrate the backend to another hosting service midtrial. The second was the identification of a bug that prevented some users from viewing the conversation. Close monitoring of the analytics framework during and after these events demonstrated minimal impact on participants’ use of the app and on the trial.

#### Computer-Mediated Technology

Findability, usability, and functionality are important concepts in information technology. Over two-thirds of respondents (199/297, 67.0%) agreed or strongly agreed that the information was easy to find within the app. A total of 83.4% (247/296) of respondents said that they found the app easy to use, and 78.0% (231/296) agreed that the visual design was appealing.

These findings were reinforced by qualitative data from the questionnaires. When asked what they liked about the app, comments about both the design and the ease of use were common. Of the 139 open-ended responses received, 23 specifically referenced the app design in a positive manner, and 31 said they liked how easy the app was to use. Comments about the design of the app covered specific features such as the graphics, the visual design, the general layout, and how well it worked:

Well designed and very engaging.

I have no interest in the points thing but I thought it was good stuff, well done. I thought the app was really well put together.

Easy to use right on your smart phone to check regularly.

In considering the collaboration of the community, when examining responses to open-ended questions asking the fathers what they liked about Milk Man, many respondents (38/139, 27.3%) made specific suggestions for improving the conversation that would better support interaction and collaboration. The most common suggestion was for the fathers to be able to start their own conversation topics, as this was not available during the trial, and fathers could only comment on researcher-generated content:

Also, changing the format of the CONVERSATION aspect to maybe allow users to create their own conversation and polls on particular topics that they might be seeking guidance or support on.

A chat section where we can start conversations or ask questions and answer each other’s questions. At the moment we can only talk about what Milk Man posts.

#### Health System Integration

Health system integration represents the larger system in which the intervention is being implemented. This was measured at the process evaluation level by examining how the app facilitated access to other services. Users used the app to access the websites of other health organizations a total of 912 times. This includes government and nongovernment health organizations. The 2 most common websites visited were the Raising Children Network (329) and the Australian Breastfeeding Association (264).

## Discussion

### Principal Findings

The findings described in this study demonstrate both the usage and user perspectives of the Milk Man mobile app. One of the strengths of this study is that app usage was not prescribed. The fathers were asked to use the app as they would use it in real life rather than, for example, being asked to spend a certain number of hours using it each week. This resulted in a wide variance in usage patterns, which is likely to reflect a real-life situation. The process evaluation provided 7 key insights:

- The Milk Man app intervention is an acceptable approach, and the weeks immediately around the time of their baby’s birth may be a key time to reach fathers with information.
- The conversation forum emerged as the hub of app activity; however, there are ways it could be strengthened.
- Push notifications were an effective way of encouraging engagement.
- The library was well received and trusted, but the fathers wanted additional and more comprehensive information.
- Gamification can be a powerful motivator for usage for some members of this target group.
- The app showed encouraging results in facilitating conversations between partners.
- Working in partnership with the app developer throughout the trial was beneficial.

### Acceptable Approach

The data show that the Milk Man app intervention was an acceptable approach and one that the fathers were prepared to engage with and received value from. User perspectives confirmed this, with high percentages of fathers agreeing that the app was easy to use, the visual design was appealing, and that they would recommend the app to other fathers. The usage data suggested that the weeks immediately before and after the birth of their baby may be a key time to reach fathers with information. Milk Man is the first breastfeeding app targeted to fathers, and the research on using digital interventions to reach new fathers is in its infancy. This finding of acceptability is important as other studies have demonstrated that while fathers are important in providing breastfeeding support to their partners, they can sometimes feel that antenatal education is not targeted to them [25,47,48].

### The Conversation Forum Was Central

There was little in the literature to suggest how fathers would use a breastfeeding conversation forum; however, the forum emerged as the focal point of the app. When asked what motivated them to use the app, *liking seeing what other dads have written* was the second highest motivator. Almost all the most read library articles and external links followed from the app originated from links within the conversation forum. Our team has previously reported how fathers have used the app to seek and offer social support [31]. This finding is consistent with that of other researchers who have reported that fathers use parenting forums to find emotional support [28], with parenting websites being particularly helpful in supporting men’s transition to fathering [49].

Whereas the conversation was clearly important, some of the overall perspectives on its value were relatively low. Although 63% of respondents said it was good hearing from other dads, only 30% found the conversation engaging, and only 30% trusted the information in the forum. There are real opportunities to explore further how this forum could best work on a population level, and participants had some suggestions for how to improve it.

The main suggestion was the ability for fathers to start their own conversation topics. Several fathers also suggested that having an active researcher participating in the conversation could be beneficial. Electronic coaching has been demonstrated as a promising approach to healthy lifestyle interventions [50], yet little is known about how this would impact a father-focused perinatal intervention. Having an active peer-facilitator embedded in the app may help to start conversations, to answer respondents, and increase opportunities for conversations and support. A higher percentage of fathers commented in the Milk Man conversation forum as compared with what has been observed in other studies [31]. Trialing the app on a population level will increase the number of fathers in each group and potentially impact the level and quality of conversation.

### Push Notifications

The push notifications proved to be an effective way of encouraging engagement with a mobile app and the analytics data show that there was increased app activity on the days that new content was added to the app and the push notifications were sent out. This was reinforced by fathers stating that the push notifications were the highest factor motivating their use of the app. Push notifications have been associated with increased engagement in other studies [51], including with a workplace health promotion intervention reporting users being more likely to engage with the app in the 24 hours after a notification was sent [52]. There is a balance to be struck as too many push notifications may be annoying and cause people to turn them off, and too few may cause people to lose connection with the intervention. The Milk Man app sent 2 notifications each week, however, more may be acceptable, particularly in the weeks immediately before and after the birth of the baby when usage was at the highest. More research is needed to understand the optimal schedule.

### Library

The use of the information in the library section was strongly associated with the conversation forum. Articles and websites that contained links from the conversation were more likely to be highly accessed. The app was useful as a gateway to other organizations. The top 2 external websites visited were national peak bodies (Raising Children Network and the Australian Breastfeeding Association) that are sources of credible and reliable information.

Fathers (79%) trusted the information in the library section, yet only 25% reported coming to the app when they needed to source information. The library contained information on topics broader than breastfeeding, including sleep, crying, fatherhood, and mental health among others. Despite this, there was a strong push for more diverse and greater quantity of content in the library. Repositioning the app to be both a breastfeeding *and* early parenting app may help with this. Recent research with new and expecting parents in Canada has highlighted the need for breastfeeding information to be provided in a broad context. [53] This needs assessment for an infant feeding website found that fathers most wanted information on the benefits of breastfeeding, identifying babies’ cues, how fathers can be involved and help their partners with breastfeeding, and what to expect in the early days.

### Gamification

Use of the gamification strategy was mixed in this study. Some fathers embraced it and it was their main motivator for using the app, whereas others reported that it had an adverse impact. There were differences in how people perceived gamification, with participants who were still using the app at 6-weeks postpartum being significantly more likely to report that the gamification functions were encouraging use, than those who stopped before 6 weeks. Other researchers have found that gamification can positively impact aspects of engagement [54], and more research is needed to better understand this dynamic in the context of Milk Man.

This study has shown that gamification can be a powerful motivator with this target group; however, care needs to be taken to better understand how its inclusion may impact those who reported not enjoying it, and the app should include the option of being fully functional without participation in the gamification.

### Conversation Between Parents

A key intent of the app was to increase parental self-efficacy by encouraging communication between parents. This, along with increasing understanding and knowledge about breastfeeding, was important in giving parents the tools to work together. Throughout the app, the content regularly suggested that fathers *check-in* with their partners about different issues. Over half of the fathers overall said that the information in the app had led to a discussion with their partner. This was more apparent for those participants who were still using the app at 6 weeks postpartum. Aiming to keep people engaged with the app for a longer period may increase the level of discussion between partners.

The findings from this study showed promising results in terms of fathers discussing or showing their partner something from the app. Research from a text messaging–based study for fathers has also found that targeted content delivered in a mobile intervention can encourage conversation between parents [55]. These are important findings as parents who work together to prepare for challenges and changes in the perinatal period fare better in terms of mental health outcomes compared with those who do not [56].

### Partnership With App Developer

Many researchers have recommended bringing app developers onboard early in the ideation process and involving them in the project planning and implementation [38,57,58]. Our project benefited significantly from implementing this recommendation, and the app developer was engaged at the design and ideation phase of the study and remained a team member throughout. In addition, the app developer contributed heavily to the app’s design and usability, both of which were key factors with many participants.

Trialing an app over a 24-month period is a long time, and there were several technological events identified over this time period, including software bugs and operating system updates. The customized analytics framework embedded in the app allowed for fine-grained monitoring of per-user use and early detection of technical issues. The issues that were identified could be addressed quickly and smoothly with minimal disruption to participants and the project implementation. Other studies have reported difficulties working with app developers or with technological challenges impacting the study implementation [59-61]. By engaging app developers as part of the research team and having them be, in part, responsible for monitoring the implementation throughout the trial, technological challenges are more likely to be identified earlier and addressed promptly.

### Recommendations

This paper adds significant understanding of on how to effectively use a mobile app to reach fathers with information during the perinatal period and has resulted in the following recommendations for future research:

- In developing mobile apps for fathers, considered engagement is key. Incorporating regular push notifications that are carefully timed and linked to new content can be an effective way of encouraging engagement with a mobile app.
- Incorporating user consultation throughout the app development process and working in partnership with app developers are important steps.
- The weeks immediately around the birth of their child are likely to be a key time when fathers are receptive to new information, and more information and support should be targeted toward fathers at this important time.
- Gamification can be a powerful motivator with this target group; however, care needs to be taken to understand how its inclusion may impact those who do not enjoy it, and apps should be fully functional without participating in the gamification.
- The Milk Man app should be released publicly in Australia to enable research into the impact and the engagement of fathers on a national level.
- To standardize and ensure best practice in app development, public health researchers should consider broad process evaluation plans. Researchers should plan ways to closely monitor the robustness of the technology over time to ensure any impact on the intervention is identified and addressed quickly.

### Strengths and Limitations

A strength of this study is the triangulation of robust and objective app usage data collected from the custom-built app analytics framework, with more subjective quantitative data collected from users via a Web-based questionnaire. The combination of both types of quantitative data was integral in understanding user involvement in the study and how that impacted on process evaluation indicators. The comprehensive approach to reporting on process evaluation provides a framework that can be adapted by other researchers. A notable limitation of this study was that not all participants completed the 6-week questionnaire, which resulted in a gap in understanding of their motivation to use the app. Having brief incidental assessments delivered through the app directly could have been one way of mitigating this loss.

A further limitation relates to the generalizability of the findings as all participants were from metropolitan Perth and were more highly educated than the general population, which may have biased the results. In addition, participants were recruited directly from antenatal classes. As these classes are recommended by care providers, but not mandated, this may have introduced a bias in that participants may already be more engaged with childbirth and breastfeeding than other members of the target group. Further research needs to be undertaken to understand the acceptability and impact of this method with people living outside of the Perth area, with Aboriginal and Torres Strait Islander parents, culturally and linguistically diverse parents, and with other specific populations.

### Conclusions

Although this paper does not report on the effectiveness of the Milk Man mobile app in relation to breastfeeding outcomes, it does provide useful insights into the effectiveness of the innovative strategies that were incorporated in the app to encourage fathers to use the app. As this was the first breastfeeding app for fathers, little was known about how participants would interact with the app, and these comprehensive results will help guide future work in this area and with this target group. There are many different aspects that can affect the implementation of health promotion interventions using a mobile app. Having a process evaluation framework that is comprehensive and specifically focused on areas that include the robustness of the technology and interaction between users and the app interface, will provide an overall picture of usability and acceptability. This was significantly aided by the custom-built analytics framework embedded in the app. Reporting process evaluation indicators against a broad evaluation framework as described here will help researchers better understand and interpret app intervention studies.

Current research in this field highlights that fathers are important in breastfeeding, that they want to help support their partners, and that they need additional information and support. This study demonstrates that fathers are prepared to seek that information and support through a carefully designed mobile app. It was hypothesized that increasing paternal support for breastfeeding may have a positive impact on breastfeeding outcomes. This paper describes the way in which fathers used the Milk Man app; the next stage is to examine the impact of this on behavior change and breastfeeding outcomes. The strategies described here show encouraging results in engaging fathers and the learnings and recommendations from this research will inform the continued development of Milk Man to better support families.

## Appendix

Multimedia Appendix 1 Milk Man library headers.

Multimedia Appendix 2 Questionnaire (Milk Man questions at 6 weeks postpartum).

## Abbreviations

PIFI: Parent Infant Feeding Initiative
RCT: randomized controlled trial
