# Supplementary material for: Gamifying Breastfeeding for Fathers: Process Evaluation of the Milk Man Mobile App
Source: JMIR Pediatr Parent. 2019 Jun 20;2(1):e12157. doi: 10.2196/12157 (PMC6716479; doi:10.2196/12157)
Supplement: Multimedia Appendix 1 [file pediatrics_v2i1e12157_app1.docx]

**Milk Man Library Headers**

1. **So, you’re going to be a dad!**
   1. Now what???
   2. Preparing for fatherhood
   3. What kind of dad will you be?
   4. How to be a dad
   5. Get your team together
   6. Time off work
   7. Supporting new dads
   8. Healthy pregnancy
   9. Smoking / alcohol in pregnancy
2. **Yeah yeah, we know, breast is best – (actually, why is that again?)**
   1. Why is breastmilk good?
   2. Why is breastfeeding good for mums?
   3. Isn’t formula just as good?
   4. Every breastfeed is a success
   5. Cost benefits
3. **Planning for Breastfeeding**
   1. Do men need to worry about breastfeeding?
   2. Consider a breastfeeding plan
   3. Look into breastfeeding antenatal classes
   4. Practically speaking…
4. **Getting it off to the breast start (see what we did there?)**
   1. What can I do to help it get off to a good start?
   2. My partner is in pain – Why? What can I do?
   3. Where is the milk!?!
   4. Hindmilk / foremilk
   5. How big is my baby’s stomach?
   6. Help, my baby is losing weight!
   7. How often should the baby feed?
   8. Is he getting enough?
   9. What about dummies and bottles?
5. **What to expect**
   1. Relationship changes
   2. Why is my baby crying?
   3. Ahem, what about sex?
   4. Feeling low?
   5. Will I ever sleep again?
   6. What is with that poo?
   7. What’s baby doing now?
   8. Say What now?!?
6. **What can I do to help?**
7. **Troubleshooting**
   1. Breastfeeding problems
   2. Attachment
   3. Insufficent supply?
   4. Nipple care
   5. Breast and nipple thrush
   6. Mastitis
   7. Engorgement
   8. Biting
8. **Bonding**
   1. Dads skin-to-skin
   2. How can I bond without feeding?
9. **Breastfeeding in public**
   1. What’s the deal, can you breastfeed in public?
   2. Can’t she just do it at home?
   3. Tips and strategies
10. **Do I really matter?**
    1. Think you can’t help with breastfeeding? Lets hear from some mums
    2. Feeling a bit on the outta?
11. **Support just for dads**
    1. DadsWA –Ngala
    2. Mensline
    3. How Is Dad Going (HIDG)?
    4. Man
    5. The Fathering Project
12. **General Parenting / Health support**
    1. Australian Breastfeeding Association
    2. Lactation consultants
    3. Pregnancy, Birth and Baby Helpline
    4. Child health nurse
    5. The BumpWA
    6. Raising Children
    7. Health direct
    8. Ngala
    9. Beyond Blue
    10. PANDA
    11. Lifeline
13. **Additional information**
    1. Alcohol and breastfeeding
    2. Going back to work
    3. Expressing and storing
    4. Mix feeding
    5. When Breastfeeding doesn’t work out
    6. Introducing solids
    7. Smoking and breastfeeding
    8. Drugs and breastfeeding
14. **Strange but true Breastfeeding facts….**
