# Supplementary material for: Gamifying Breastfeeding for Fathers: Process Evaluation of the Milk Man Mobile App
Source: JMIR Pediatr Parent. 2019 Jun 20;2(1):e12157. doi: 10.2196/12157 (PMC6716479; doi:10.2196/12157)
Supplement: Multimedia Appendix 2 [file pediatrics_v2i1e12157_app2.docx]

**The following questions are from the six week questionnaire that were asked of fathers in the Milk Man app groups**

**As part of this study you have been given access to the Milk Man app. The following questions refer to the app.**

1. Have you installed the Milk Man app?
   1. Yes / No
      1. If yes, (go to 2)
      2. If no, why not? Please let us know why not, (tick all that apply)

I don’t use apps

I can’t be bothered looking at it

I’m too busy

I don't trust info from apps

I don’t like the look of it

Breastfeeding isn’t men’s business

Embarrassing having it on my phone

Just haven’t gotten around to it

Partner not breastfeeding

Other ____

- - 1. Do you want us to contact you to help you install it?
       1. Yes / No
          1. Yes, please provide contact number (go to end)
          2. No (go to end)

1. Have you used the app?
   1. Yes
      1. Are you still using it
         1. Yes (go to 3)
         2. No
   2. No Go to 2bi
      1. If no, do you intend to?
         1. Yes (go to end)
         2. No (go to 2bi.2.a)
            1. Please let us know why not, (tick all that apply)

I don’t use apps

I can’t be bothered looking at it

I’m too busy

I don't trust information from apps

I don’t like the look of it

Breastfeeding is not men’s business

It’s embarrassing having it on my phone

My partner isn’t breastfeeding

Other ____ (go to end)

1. **What motivates you to visit the app? Please tick all that apply**
2. The push notifications remind me to check in
3. I need to find information
4. I want to get points
5. I like seeing what other dads have written
6. I like the conversation topics
7. I like that there’s always new stuff to read
8. I need to find a service or organisation
9. I like the competition element

**4. These questions relate to the LIBRARY section**

Please indicate how strongly you agree or disagree with the following statements.

|  | Strongly Agree | Agree | Don’t know | Disagree | Strongly disagree |
| --- | --- | --- | --- | --- | --- |
| The information in the library is easy to find |  |  |  |  |  |
| There is enough information |  |  |  |  |  |
| I am learning new information |  |  |  |  |  |
| I trust the information contained in the library |  |  |  |  |  |
| The links to further information are appropriate and useful |  |  |  |  |  |
| I come to the app when I needed to find information |  |  |  |  |  |

**5. These questions relate to the CONVERSATION section**

Please indicate how strongly you agree or disagree with the following statements.

|  | Strongly Agree | Agree | Don’t know | Disagree | Strongly disagree |
| --- | --- | --- | --- | --- | --- |
| I find the conversation engaging |  |  |  |  |  |
| It is good hearing from other dads |  |  |  |  |  |
| I sometimes return to the conversation to see if there are any new comments |  |  |  |  |  |
| I trust the information in the conversation |  |  |  |  |  |
| I sometimes check back to see if my comment had received any upvotes |  |  |  |  |  |
| Getting upvotes encourages me to comment more |  |  |  |  |  |
| I have acted on advice I have read in the conversation |  |  |  |  |  |
| I have discussed something with my partner that I read in the conversation |  |  |  |  |  |

**6. These questions relate to the GAME**

Please indicate how strongly you agree or disagree with the following statements.

|  | Strongly Agree | Agree | Don’t know | Disagree | Strongly disagree |
| --- | --- | --- | --- | --- | --- |
| Earning points encourages me to keep using the app |  |  |  |  |  |
| Earning badges encourages me to keep using the app |  |  |  |  |  |
| My position on the leaderboard encourages me to keep using the app |  |  |  |  |  |

**7. These questions relate to MILK MAN in general**

|  | Strongly Agree | Agree | Don’t know | Disagree | Strongly disagree |
| --- | --- | --- | --- | --- | --- |
| The app is easy to use |  |  |  |  |  |
| The visual design of the app is appealing |  |  |  |  |  |
| I would recommend this app to other new or expectant dads |  |  |  |  |  |
| I find the app interesting / fun to use |  |  |  |  |  |
| The app has made me more aware of how I can help with breastfeeding |  |  |  |  |  |
| Information within the app has lead to discussions with my partner. |  |  |  |  |  |

8. What did you like about the app? (open ended)

9. What suggestions do you have to improve the app? (open ended)
